# Prevalence of bovine genital campylobacteriosis and trichomonosis of bulls in northern Nigeria

**DOI:** 10.1186/1751-0147-55-56

**Published:** 2013-08-09

**Authors:** Hassan M Mai, Peter C Irons, Junaidu Kabir, Peter N Thompson

**Affiliations:** 1 Department of Production Animal Studies, Faculty of Veterinary Science, University of Pretoria, Private Bag X04, Onderstepoort 0110, South Africa; 2 Animal Production Programme, School of Agriculture and Agricultural Technology, Abubakar Tafawa Balewa University, P. M. B. 0248, Bauchi 740001, Nigeria; 3 Department of Veterinary Public Health and Preventive Medicine, Ahmadu Bello University, Zaria, Nigeria

**Keywords:** Bovine, Brucellosis, Campylobacteriosis, Nigeria, Preputial samples, Trichomonosis

## Abstract

**Background:**

A survey was conducted to determine the prevalence of campylobacteriosis and trichomonosis, and their concurrence with brucellosis, in cattle in three states of northern Nigeria.

**Methods:**

A total of 602 preputial samples was collected from bulls in 250 herds and tested using culture and identification. Various indigenous and exotic breeds were studied and four major management systems were encountered. Age of the cattle was estimated using dentition, farm records or cornual rings.

**Results:**

The estimated true animal-level prevalence of *Campylobacter fetus* infection was 16.4% (95% CI: 13.0-20.7), of which 18.5% was *C. f. fetus* and 81.5% was *C. f. venerealis*. Of the latter, 92% were *C. f. venerealis* biovar intermedius strains. Animal-level prevalences in Adamawa, Kano and Kaduna states were 31.8%, 11.6% and 8.3% respectively, and were highest in bulls >7 years old (33.4%) and in the Gudali breed (28.8%). Of the 250 herds, 78 (25.5%, 95% CI: 19.4-32.7) had at least one infected bull, and herd prevalence was highest in the pastoral management system (43.5%). After adjustment for confounding using multivariable analysis, the odds of *C. fetus* infection were highest in Adamawa state (*P* < 0.01), in the pastoral management system (*P* < 0.01), and in bulls >7 years old (*P* = 0.01), and tended to be higher in *Bos taurus* breeds (*P* = 0.06). There was a strong positive association between the presence of campylobacteriosis and brucellosis (*P* < 0.01), both within bulls (OR = 8.3) and within herds (OR = 16.0). Trichomonosis was not detected in any herds.

**Conclusion:**

Bovine genital campylobacteriosis is prevalent particularly in the pastoral management system in northern Nigeria, with *C. f. venerealis* biovar intermedius as the major aetiology. There was a strong positive correlation between the occurrence of campylobacteriosis and brucellosis. No evidence of trichomonosis was found in herds in this study.

## Background

Bovine genital campylobacteriosis (BGC) and trichomonosis are economically important venereal diseases that occur worldwide and are characterized by infertility, embryo mortality, abortion, irregular oestrous cycles and long calving intervals [1-4]. The diseases tend to occur in areas with extensive cattle management and natural breeding [5-7]. Pregnancy rate in BGC can be as low as 20% and abortion rate as high as 10% [8]. Sterility may occur in up to 11% of infected heifers [5]. Trichomonosis is also associated with mild to severe pyometra, low birth weight and decreased calf crop of up to 50% [6,9].

The most common cause of BGC is *Campylobacter fetus venerealis*[7]. Bulls carry *C. f. venerealis* subclinically in their prepuce, and older bulls above three years may remain permanently infected and also serve as source of infection through the use of semen from infected bulls in artificial insemination (AI) programmes [2,10]. Sub-speciation of *C. f. venerealis* has been an area of some interest and there is an unusually high prevalence of *C. f. venerealis* biovar intermedius in South Africa [11]; however, the prevalence of this isolate has not been studied in Nigeria. *Campylobacter f. fetus*, found in the genital and intestinal tracts of cattle and sheep [2,10], is also a cause of infectious infertility in cattle and is also reported to cause a wide variety of invasive diseases in humans [12].

A survey of *C. fetus* in a communal grazing area in South Africa showed a high prevalence of 28.7% [13], and 40-47% of dairy cows were found to be seropositive in one study in the USA [14]. Some field studies have been done in Nigeria [4,15-18], reporting animal-level prevalences of 2.9-11% and herd-level prevalences of 20-22%; however, these were done on few herds and in limited locations, and in some cases targeted animals with a history of reproductive failure. The occurrence of BGC is believed to be grossly under-estimated and under-reported in Africa [7,10].

Trichomonosis is caused by *Tritrichomonas foetus*. Bulls are primary reservoirs of *T. foetus* whereas cows clear the infection spontaneously. Strong evidence exists that bulls older than 4 years rarely recover spontaneously and are long-term carriers [19]. Transmission occurs during natural mating; however, infection affects neither semen quality nor sexual behaviour [20]. Semen used in AI may also be a source of transmission, as the organism can survive the standard AI processing methods [21]. Bull infection rates range from relatively low (0-4%) in some herds to high (27-33%) in others [5,22,23].

Estimates of annual losses to the USA beef industry as a result of trichomonosis have been as high as US$ 650 million [24]. Despite the potentially huge economic losses due to infertility, cost of culling and replacement, and cost of treatment [23], the prevalence and impact of this disease in Nigeria is largely unknown. The only studies conducted on *T. foetus* in Nigeria were by Adeyeye *et al*. [25], Akinboade [26] and Ayoade *et al.*[27] who reported prevalences of 0%, 14.9% and 100% respectively.

There have been no studies on the concurrence of campylobacteriosis, trichomonosis and brucellosis in Nigeria, nor any recent studies covering different states and production systems. Concurrent herd infection with trichomonosis and *C. f. venerealis* in bulls was shown to be common in South Africa [13] and northern Australia [5]. Concurrent infection with *C. fetus* and *T. foetus* has been reported to cause reproductive failure in camels [28]. However, there is no evidence to suggest that infection with one agent predisposes to infection with the other agent [5]. Determining the extent of concurrence of diseases is a useful tool in devising control strategies likely to impact on both diseases.

Continuous movement of cattle and sharing of bulls is practiced in Nigeria, putting many herds at risk of venereal infections. Over the years both governmental and non-governmental interventions in the livestock sector have imported semen for AI programmes; however, there is no information on the screening of AI semen for *C. fetus, T. foetus* or *Brucella abortus* in Nigeria. The desire of the Nigerian government to promote dairy production and the upsurge of commercial dairy production through the use of AI programmes to upgrade Nigerian indigenous cattle, without proper screening of semen for infectious reproductive diseases, are of concern to the livestock industry. Therefore, this study was designed in order to determine the animal- and herd-level prevalences of campylobacteriosis and trichomonosis in mature bulls in cattle herds in three important cattle producing states of northern Nigeria, as well as the concurrence of the two infections with each other and with *B. abortus* infection.

## Materials and methods

The research protocol for this study was approved by the Animal Use and Care Committee and the Research Committee of the University of Pretoria (Protocol no. V073-08).

### Sampling area and sample size

**Figure 1:**
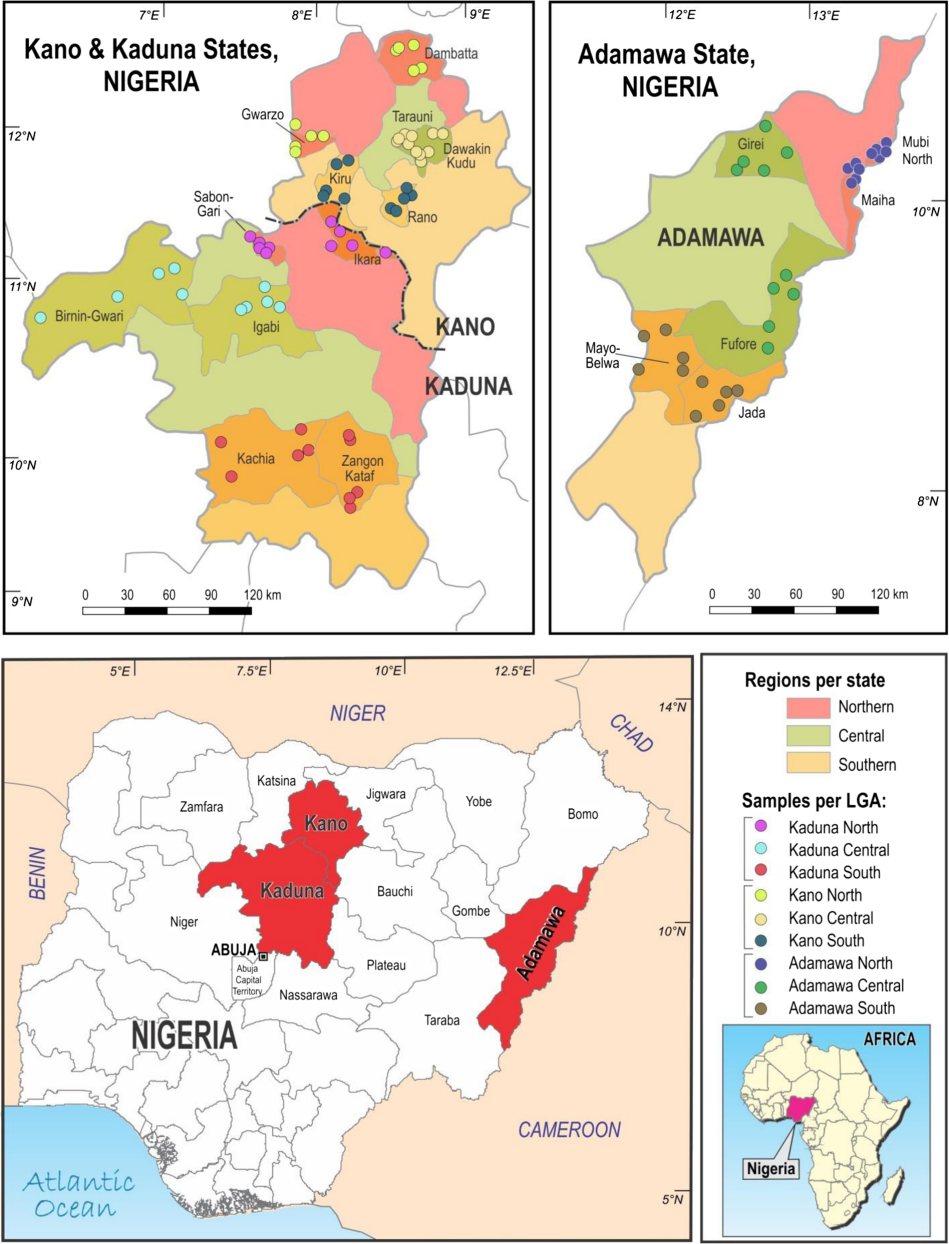
Map of Nigeria showing the three states, 18 local government areas and 89 wards sampled in northern Nigeria.

Three states (Kaduna, Kano and Adamawa) out of the 19 northern states of Nigeria were sampled (Figure 1). The selection was based on their location, proximity to a reliable laboratory, farming systems, human and animal populations, cooperation from farmers, sharing of international borders and variety of animal breeds.

In order to estimate the herd-level prevalence of campylobacteriosis and trichomonosis, a multistage sampling strategy was used. Assuming an estimated herd prevalence (*P*_exp_) of 20% for both diseases, an absolute allowable error (*d*) of 7.5% and a confidence level of 95%, the formula *n* = 1.96^2^*P_exp_*(1 – *P*_exp_)/*d*^2^[29] was used to calculate a required sample size of 110 herds using simple random sampling. A low intra-cluster correlation coefficient of *ρ* = 0.1 was used to account for geographic clustering within wards and local government areas (LGAs), resulting in a design effect (*D*) of 2.2. This was calculated using the formula D = 1 + (b – 1)*roh [30], where *b* is the average number of samples per cluster, in this case approximately 13 farms per LGA, and *roh* is the rate of homogeneity, equivalent to the intra-cluster correlation coefficient (*ρ*) in single-stage cluster sampling. This resulted in a required sample size of 242 herds. To ensure even distribution across states, each state was divided into 3 geographic zones based on administrative divisions. In each zone, two LGAs were randomly selected, giving a total of six LGAs per state (Figure 1). On average in each LGA, 5 wards were randomly selected, and in each ward 3 herds were sampled, giving 271 herds in total, of which 250 contained bulls eligible for sampling. An infected herd was defined as one in which one or more *C. fetus* or *T. foetus* infected bulls were detected.

### Sampling for *Campylobacter fetus* and *Tritrichomonas foetus*

All post-pubertal bulls present in selected herds were sampled once for both *C. fetus* and *T. foetus* by collecting preputial samples using preputial scraping technique as follows: The prepuce was cleaned with paper towel and the hair around the preputial orifice clipped where necessary. The samples were collected by insertion of a 53-cm rigid AI pipette attached to a sterile 20 ml disposable hypodermic syringe, through the preputial orifice to the fornix of the prepuce. The preputial lining and *glans penis* were scraped vigorously for about 45 seconds while applying vacuum with the plunger of the syringe as described by Irons *et al*. [2]. The pipette was withdrawn gradually while still applying negative pressure to the plunger. For *C. fetus*, the sample was directly inoculated in Mueller Hinton broth (Oxoid, CM0405B) for transportation to the laboratory for isolation, while for *T. foetus*, the same sample was inoculated into a combined transport and culture media as described below. In addition, a drop of the fresh sample was put on a clean glass slide, a cover slip was placed on the sample, and it was viewed directly using a normal bright field microscope.

### Isolation of *Campylobacter fetus* from bulls

Preputial samples were streaked out using a 25 μl loop onto the surface of Columbia agar base (Oxoid, CM0331B) containing *Campylobacter* Skirrow’s supplement (Oxoid SR0069E), 7% citrated/defibrinated blood, polymixin B, trimethoprim, vancomycin and amphotericin B and blood agar without antibiotics. Plates were incubated in a hydrogen enriched microaerophilic atmosphere consisting of 10% CO_2_, 10% H_2_, and 80% N_2_ using *Campylobacter* system Gas Generating Kit (Oxoid, BR0060A) with palladium catalyst at 37°C for 72 hours, shielded from light. At 72 h, a representative of a dewdrop colony which was smooth, shiny and grey to pink in colour with organisms that were Gram-negative, vibroid in shape, oxidase- and catalase-positive was transferred to blood agar base (Oxoid, CM0055), streaked for purity and incubated under the same conditions as the sample described above. Each culture and incubation run was verified by using control strains of *C. f. fetus* and *C. f. venerealis* (ATCC 33247 and 19438 respectively). The identification of each colony obtained was confirmed using the Dry spot latex agglutination test (Oxoid, DR150M), according to manufacturer’s instructions.

The isolates were subjected to biochemical testing for H_2_S production using TSI agar (Oxoid, CM0277B), for aerobic growth, for growth at 25°C and 42°C and in the presence of 1% glycine and 3.5% NaCl, and for sensitivity to cephalothin and nalidixic acid. *Campylobacter fetus* grew at 25°C, did not produce H_2_S using TSI agar, was sensitive to cephalothin but resistant to nalidixic acid, and was oxidase and catalase positive. Two phenotyping tests, i.e. tolerance to 1% glycine and H_2_S production using lead acetate paper [10,11], were used to differentiate the subspecies. *Campylobacter f. fetus* grew on 1% glycine medium and produced H_2_S; *C. f. venerealis* did not grow on 1% glycine and did not produce H_2_S; *C. f. venerealis* biovar intermedius did not grow on 1% glycine but produced H_2_S in L-cysteine supplemented “sensitive medium” [11].

### Isolation of *Tritrichomonas foetus* from bulls

For logistical reasons, two methods of isolation were used in this study; however, they were considered equal. The first 300 preputial samples were each directly inoculated into a commercial transport and field culture kit (InPouch™ TF system; Biomed Diagnostics, San Jose, CA, USA) that allows for growth of the trichomonads and direct microscopic examination without aspiration of the inoculums. The last 302 samples were each placed in a sterile Whirl-Pak® bag containing 10 ml of "*Trichomonas* medium" enriched with heat-inactivated foetal calf serum (CN 3332 Highveld Biological, Lyndhurst, South Africa) (80 ml/l medium) and 2 ml chloramphenicol/l (CAPS Pharmaceuticals, South Africa). The medium was examined directly through the plastic pouch in the field under a standard light microscope using a magnification of 100 or more for the presence of motile protozoa with three flagella. The pouch was incubated at 37°C and examined every 24 h for 7 days. In addition, the prepared enriched "*Trichomonas* medium" was dispensed aseptically into sterile McCartney bottles in 10 ml aliquots and used as both transport and culture medium for all the samples collected for isolation of *T. foetus* with microscopic examination of the medium at intervals from day 1 to day 7 after inoculation, taken from the bottom of the bottle. For wet preparation or direct microscopy, a drop of inoculated sample was put on a clean glass slide with a cover slip for immediate observation under the microscope at ×10 and ×40 magnification. The result was recorded as positive when trichomonad organisms displaying unique morphological characteristics were present, or negative if there was no growth of trichomonads.

### Other data collected

Herds were categorized into the four main management systems as follows: Pastoral herds are those in which the cattle graze communally during the rainy season, but may cover large distances in search of pasture and water during the critical period of the dry season. Natural pasture is the major source of feed for livestock in the pastoral system. Agro-pastoral herds are herds that go out for communal grazing in the morning and return in the evening without covering large distances but in addition supplementary feeds are provided. The farmers are involved in agricultural activities and use crop residues for their livestock. Commercial farms are usually organized and fenced farms with paddocked, improved pasture and a regular supply of supplementary feeds. Zero-grazing cattle are tethered and feed is constantly supplied to them.

The breed of each animal was recorded in the following categories: Bunaji, Gudali (Adamawa Gudali and Sokoto Gudali), other *Bos indicus* (Rahaji, Wadara, Ndama and Brahman), *Bos taurus* (Friesian, Simmental, Jersey and Brown Swiss) and *B. indicus* × *B. taurus* crosses. Age was estimated using farm records, dentition and, in some cases, cornual rings.

As part of a separate study [31], all the bulls sampled in this study were also tested for brucellosis using the Rose-Bengal plate-agglutination test, with confirmation using a competitive enzyme-linked immunosorbent assay kit (COMPELISA, VLA, Weybridge, UK).

### Data analysis

All statistical analyses were done using Stata 12 (Stata Corporation, College Station, TX, USA). The prevalences of campylobacteriosis and trichomonosis, overall and within states, age, breed and management systems were estimated both at animal and herdlevels, taking into account sampling weights in the multistage survey design. For each ward the sampling fraction was calculated as the product of the proportion of wards sampled within each LGA and the proportion of LGAs sampled within each state. The sampling weight was then calculated as the inverse of the sampling fraction. Since it was not possible to calculate the proportion of farms sampled within each ward, but all eligible animals on each farm were sampled, the same sampling weight was assigned to every animal within a ward. Sampling weights were then used to weight the contribution of each bull’s test result to the overall prevalence and to adjust the standard error to account for clustering, using the ‘*svy*’ commands of Stata 12.

Prevalence estimates were then compared using the Chi-square test, corrected for the survey design using the second-order correction of Rao and Scott [32]. At the animal level, true prevalence (TP) was then calculated by correcting the apparent prevalence point estimates and confidence limits using the formula described by Rogan and Gladen [33]: TP = (AP + Sp – 1) / (Se + Sp – 1), where AP = apparent prevalence, Se = sensitivity, Sp = specificity. The values used for sensitivity and specificity of the culture system for detection of *C. fetus* were 94.0% [34] and 100% [35] respectively, and for *T. foetus* detection they were 76.0% and 98.5% respectively [36].

The association of animal-level *C. fetus* infection with age, breed and management system was then analysed using a multivariable logistic regression model with state as a fixed effect and LGA, ward and herd as nested random effects. The concurrent presence of brucellosis was assessed at both the animal and herd level using the chi-square test and the strength of association estimated using the odds ratio.

## Results

### Herd-level prevalence of campylobacteriosis

A total of 250 herds containing 602 bulls, with between 1 and 24 bulls per herd (median: 1; interquartile range: 1, 2) were sampled, with 78 herds containing at least one bull positive for *C. fetus*, giving a herd-level prevalence, adjusted for sampling weights, of 25.5% (95% CI: 19.4-32.7). Herd-level prevalence was highest in Adamawa state (*P* < 0.001) (Table 1), and varied between management systems (*P* = 0.03), being higher in pastoral (43.5%, 95% CI: 24.7-64.4) than in agro-pastoral (17.4%, 95% CI: 10.8-26.8) systems (*P* = 0.01).

**Table 1:** **Prevalence of herds with *Campylobacter fetus*-positive bulls in three states of northern Nigeria, adjusted for sampling weights**

**State**	**Herds sampled**	**Negative**	**Cff**	**Cfv**	**Herds with Cff & Cfv**	**Total positive**	**95% CI**
Adamawa	94	44	10	45	5	50 (51.3%)^a^	37.0, 65.4
Kaduna	93	74	6	14	1	19 (20.7%)^b^	14.5, 28.7
Kano	63	54	2	8	1	9 (14.3%)^b^	6.8, 29.2
Total	250	172 (74.5%)	18 (6.3%)	67 (21.7%)	7 (2.5%)	78 (25.5%)	19.4, 32.7

^a,b^Values with different superscripts are significantly different (*P* < 0.01).

Key:

*CffCampylobacter fetus fetus*.

*CfvCampylobacter fetus venerealis*.

### Animal-level prevalence of campylobacteriosis

Of the 602 bulls tested, 108 were positive for *C. fetus*; the animal-level prevalence, adjusted for the survey design and test sensitivity and specificity, was 16.4% (95% CI: 13.0-20.7) (Table 2). There was a significant increase in prevalence of *C. fetus* infection with increasing age, with bulls >7 years having the highest prevalence (Table 3), also seen in the multivariable model (Table 4). Infection was apparently most prevalent in the Gudali breed (Table 3). However, after adjustment for state, management system and age, the odds of infection tended to be highest in *Bos taurus* breeds (Table 4). Crude animal-level prevalence of *C. fetus* did not differ significantly between the management systems (Table 3). However, after adjustment for state, age and breed, the odds of a bull being positive were significantly higher for pastoral systems than for zero-grazing (*P* = 0.001) and agro-pastoral (*P* < 0.001) systems (Table 4).

**Table 2:** **Prevalence of *Campylobacter *spp. in individual bulls from three states in northern Nigeria, adjusted for sampling weights and for test sensitivity and specificity**

**State**		**No. of positive bulls**		**Total positive for**	**95% CI**
	**No. sampled**	***C. fetus fetus***	***C. fetus venerealis***	***Campylobacter *spp.**	
Adamawa	235	12	63	75 (31.8%)^a^	23.3, 41.9
Kaduna	257	6	15	21 (8.3%)^b^	5.9, 12.4
Kano	110	2	10	12 (11.6%)^b^	7.6, 17.2
Overall	602	20 (3.2%)	88 (13.3%)	108 (16.4%)	13.0, 20.7

^a,b^Values with different superscripts are significantly different (*P* < 0.01).

**Table 3:** **Animal-level prevalence of *Campylobacter fetus *in bulls in three northern states of Nigeria by management system, age and breed, adjusted for sampling weights and test sensitivity and specificity**

**Variable**	**n**	**% positive**	**95% CI**
Management system			
Zero-grazing	68	17.6	12.6, 23.9
Commercial	31	24.7	9.9, 50.1
Agro-pastoral	347	13.5	8.4, 21.0
Pastoral	156	22.2	14.8, 32.0
Age			
<4 years	23	8.8	1.6, 36.2
4-5 years	339	13.6^a^	10.4, 17.7
5-7 years	200	18.7	12.1, 27.9
>7 years	30	33.4^b^	16.6, 56.3
Breed			
Bunaji	344	11.5^c^	8.1, 16.1
Gudali	149	28.8^d^	19.8, 39.9
*Bos taurus*	28	17.8	5.2, 46.7
*B. taurus* × *B. indicus*	31	18.1	3.5, 58.6
Other *B. indicus*	50	16.4	7.3, 32.9

^ab,cd^Values with different superscripts differ significantly (ab: *P* = 0.02; cd: *P* < 0.01).

**Table 4:** **Associations of state, management system, age and breed with *Campylobacter fetus *infection in bulls in three northern states of Nigeria: results of a multivariable logistic regression model**

**Variable**	**Odds ratio**	**95% CI**	***P***
State			
Kaduna	1^*^	–	–
Adamawa	10.1	4.0, 25.5	<0.01
Kano	1.3	0.5, 3.5	0.61
Management system			
Zero-grazing	1^*^	–	–
Commercial	1.9	0.3, 10.8	0.47
Agro-pastoral	1.5	0.5, 4.6	0.44
Pastoral	7.3	2.2, 24.3	<0.01
Age			
<4 years	0.9	0.2, 4.6	0.91
4-5 years	1^*^	–	–
5-7 years	1.3	0.7, 2.2	0.42
>7 years	3.4	1.3, 8.7	0.01
Breed			
Bunaji	1^*^	–	–
Gudali	1.4	0.7, 3.1	0.34
*Bos taurus*	4.2	0.9, 18.9	0.06
*B. taurus* × *B. indicus*	1.6	0.4, 6.6	0.51
Other *B. indicus*	1.1	0.4, 3.3	0.81

^*^Reference level.

### Distribution of *C. f. fetus*, *C. f. venerealis* and *C. f. venerealis* biovar intermedius strains

In all states, *C. f. venerealis* was isolated more frequently than *C. f. fetus*. The distribution of *C. f. fetus*, *C. f. venerealis* and both subspecies in herds and individual bulls in the various states are shown in Tables 1 and 2 respectively. Of the positive herds, 76.9% had *C. f. venerealis* alone, 14.1% had *C. f. fetus* alone and 9.0% had both *C. f. venerealis* and *C. f. fetus*. Of the 88 *C. f. venerealis* isolates, 81 (92%) were *C. f. venerealis* biovar intermedius strains. Both *C. f. fetus* and *C. f. venerealis* were never found together in the same bull. Both *C. f. fetus* and *C. f. venerealis* were found more in herds with bulls >7 years (7.5% and 27.5%) than bulls <4 years (0% and 8.3%) respectively. The prevalence of *C. f. fetus* and *C. f. venerealis* were higher in zero-grazing herds (25.0% and 62.5%), followed by pastoral herds (10.5% and 38.2%), commercial herds (13.0% and 17.4%) and agro-pastoral herds (3.5% and 20.3%) respectively.

### Association between campylobacteriosis and brucellosis

There was a significant positive association between the occurrence of campylobacteriosis and brucellosis both at animal-level and at herd-level (Table 5).

**Table 5:** Concurrent occurrence of campylobacteriosis and brucellosis at animal and herd-level in cattle herds from three states in northern Nigeria

		**Campylobacteriosis**		
		**+**	**–**	**Total**
*Animal-level (bulls)*				
	Brucellosis +	81	131	212
	Brucellosis –	27	363	390
	Total	108	494	602
Odds ratio = 8.3 (95% CI: 5.2, 13.4), χ^2^ = 91.3, *P* < 0.0001				
*Herd-level*				
	Brucellosis +	76	121	197
	Brucellosis –	2	51	53
	Total	78	172	250
Odds ratio = 16.0 (95% CI: 3.8, 67.7), χ^2^ = 23.6, *P* < 0.0001				

### Prevalence of trichomonosis

Trichomonosis was not isolated from any of the bulls in this study. The upper 95% confidence limit for the prevalence of positive test results in the population was 0.50%. The absence of any positive tests suggested that the specificity of the test was greater than the 98.5% reported by Cobo *et al.*[36]; therefore, adjusting for a test sensitivity of 76% and specificity of 100%, an upper 95% confidence limit of 0.65% was calculated for the true animal-level prevalence of trichomonosis in the population.

## Discussion

In the recent past, most emphasis has been placed on brucellosis as the most prominent reproductive disease of cattle in Nigeria. However, it is evident from the result of this study that *C. fetus* is common in northern Nigeria and may be an important cause of infertility. A similar study performed in Nigeria 20 years ago reported a lower prevalence of 2.9% of cattle and 20% of herds [4]. Although the authors sampled both bulls and cows, the herd-level prevalence was comparable to our study; however, the animal-level prevalence was lower, suggesting that there has been an increase in the prevalence of this disease.

An animal-level *C. fetus* prevalence of 10-15% with a herd-level prevalence of 53.8% was reported in Malawi [37] and animal-level prevalences of between 29% and 47% have been reported in other countries [13,14]. The proximity of Adamawa state to the Cameroon border may contribute to the high prevalence in the state; Mshelia *et al*. [7,17] reported a high incidence of BGC in Nigeria, where natural breeding of cattle is widely practiced, and implicated mass cattle movement across the borders of Nigeria as a major risk factor.

The crude association between breed and *C. fetus* prevalence, with the highest prevalence in Gudali bulls, disappeared after adjustment for state, management system and age. This was likely due to confounding by state, since Gudali bulls were encountered predominantly in Adamawa state which has a higher prevalence. In fact, after adjustment for state, the odds of being *C. fetus* positive tended to be higher for *Bos taurus* bulls than for Gudali bulls. It is possible that *Bos taurus* may be more susceptible than indigenous breeds to campylobacteriosis. Klastrup and Halliwell [37] also found more of the disease in exotic than indigenous cattle. However, further studies including adjustment for other possible confounders are required to be able to determine breed susceptibility.

The strong association between *C. fetus* infection and the pastoral management system, revealed in the multivariable analysis, was initially confounded by state, since more of the pastoral herds were found in the lower prevalence Kaduna state than in Adamawa state. Comingling, sharing of grazing land and common watering points, which are common in northern Nigeria, contribute to the spread of BGC. A prevalence of 28.7% was reported in communal grazing areas in South Africa [13]. Trespassing bulls from neighboring herds may double the risk of a herd being positive to BGC [38], and transmission in cattle is known to be associated with sharing, renting or mixing of bulls and other cattle on common grazing land or extensive cattle management [5,8,38].

The prevalence of BGC was higher in older bulls. This is in agreement with other reports [2,39]. The retention of infection in older bulls than the younger bulls may be due to the increase in number and size of the irregular crypts in the epithelium of the penis and persistent colonization of the lower reproductive tract of mature bulls by *C. fetus*[39]*.* However, McCool *et al.*[5] and Swai *et al*. [40] observed that both younger and older bulls could remain carriers after infection; this requires further investigation.

Of the two subspecies of *C. fetus*, *C. f. venerealis* was more common, which is in agreement with other studies [2,4,8]. It is noteworthy that *C. f. venerealis* is more pathogenic than *C. f. fetus*[10,41]. This is the first report of *C. fetus* subsp. *venerealis* biovar intermedius in Nigeria. This has also been reported from South Africa [11]. The latter study also demonstrated the incorrect classification based on subspecies-specific PCR assays, making phenotypic characterization following bacterial culture superior to PCR assays for identification of *C. fetus* subspecies*.* In a genomic analysis of *C. fetus* subspecies, two assays were specific for *C. fetus* subsp. *venerealis* AZUL-94 strain, with a further single assay specific for the AZUL-94 strain and *C. fetus* subsp. *venerealis* biovar intermedius in Australia [42]. The assays developed in the genomic analysis of *C. fetus* subspecies highlight the complexity of targeting strain specific virulence genes for field studies for the molecular identification and epidemiology of *C. fetus*[42].

The strong association between the occurrences of campylobacteriosis and brucellosis suggests that, although their major mode of transmission is different, they share some common risk factors. The separate study on brucellosis using the same bulls [31] showed that the infection was more prevalent in older bulls and in the pastoral management system, and half of all the bulls that had hygroma, a common manifestation of brucellosis, also had BGC. This is consistent with the findings of Zhao *et al*. [41], who reported some degree of mixed infections of campylobacteriosis and bovine brucellosis. Further investigation of risk factors for the two diseases would be useful in order to inform control measures for both diseases. Another possible explanation may be that one infection predisposes an animal to the other infection, possibly due to an effect on the immune system. Neta *et al*. [43] reported that *B. abortus* invades phagocytic and non-phagocytic host cells of cattle, inhibiting phagosome-lysosome fusion thereby affecting the innate and adaptive immunity against brucellosis. Such cattle may also be susceptible to campylobacteriosis.

*Tritrichomonas foetus* was not isolated from any of the bulls sampled in this study despite using an efficient diagnostic protocol, including a gold standard test [21], and a commercial transport and field culture kit, with a sensitivity of up to 100% [44] on half of the samples. Even assuming a test sensitivity of only 76%, we could be 95% certain that the true prevalence of trichomonosis in the area was below 0.65%. Considering the extent of bull sharing and comingling of cattle in our study area, it would be expected that, were the infection present, the true prevalence would be far greater than 0.65%, therefore it is probable that the disease is absent in our study area. This finding is in agreement with surveys conducted in 384 bulls in Malawi [37]; in 58 bulls in Tanzania [40] and most recently in 299 males, 101 female cattle and 3 abomasal and foetal fluids in northern Nigeria [25]. It therefore suggests that trichomonosis may not be a significant problem in northern Nigeria despite reports of its occurrence in southern Nigeria [26,27]. Breed and ecological differences may be responsible for the variation in disease occurrence in northern and southern Nigeria. Most of the breeds in the south are the short and humpless Ndama, Muturu and Keteku; while the climate is relatively hot with high humidity as opposed to the relatively cool and dry northern parts. Very recently it was shown that *T. foetus* may transform into pseudocysts or endoflagellar form because of adverse environmental conditions or presence of drugs [45] and may therefore not be detected.

## Conclusion

Bovine genital campylobacteriosis should be considered in all investigations of herd infertility problems in northern Nigeria, since more than 50% of herds are infected in some areas. The risk of *C. fetus* infection is highest in the pastoral management system usually practiced by the Fulanis in northern Nigeria. Consideration should therefore be given to recommending alternative management systems, such as the agro-pastoral system, which carries a lower risk, and specific preventative measures such as vaccination. Campylobacteriosis and brucellosis often occur together in herds, and may share similar risk factors; therefore control measures can be instituted to target both diseases.

## Competing interest

The authors declare that they have no competing interests.

## Authors’ contributions

HMM initiated and designed the study, was responsible for herd visits, collection, planning of data, analysis and interpretation of data and writing the draft manuscript. PNT was involved in the design of the project, performed the statistical analysis and interpretation, and critical revision of the paper. PCI also took part in the planning of the study and proof reading the article. JK was involved in the initial data input, analysis, interpretation and revision of the manuscript. All authors have read and approved the final manuscript.
